# Impact of Chronic Infection‐Induced Inflammation on TREG Cell Homeostasis

**DOI:** 10.1002/eji.70265

**Published:** 2026-08-20

**Authors:** Zachary R. Lanzar, Daniel L. Aldridge, Julia N. Eberhard, Elisa Cruz‐Morales, Isabella O. Conway, Molly E. Bunkofske, Joseph A. Pereira, Melissa Parker, Chryssa Kanellopoulou, Christopher A. Hunter

**Affiliations:** ^1^ Department of Pathobiology University of Pennsylvania Philadelphia PA USA; ^2^ Department of Immunology Blavatnik Institute, Harvard Medical School Boston MA USA; ^3^ Incyte Research Institute Incyte Corporation Wilmington DE USA

**Keywords:** infection, inflammation, Treg

## Abstract

Thymically‐derived regulatory T (Treg) cells express the transcription factor Foxp3 and are maintained at homeostasis by the cytokine IL‐2. These specialized cells mediate peripheral tolerance and determine the balance between protective and pathological T cell responses during infection. Acute infection of mice with *Toxoplasma gondii* results in thymic atrophy and reduced production of IL‐2 that correlate with a “crash” in Treg cell populations. Here, kinetic studies of mice infected with *T. gondii* reveal that as acute infection resolves, there is a restoration of IL‐2 and reversal of thymic hypertrophy associated with partial restoration of the Treg compartment. Fate‐mapping of the Treg cell compartment showed that infection‐induced thymic involution did not severely impact the ability to generate Treg cells. Moreover, the peripheral Treg cells that survived the infection‐induced “crash” were the major contributors to the rebuilt Treg compartment. However, the use of antiparasitic drugs to reduce chronic parasite replication and inflammation improved generation of thymic Treg cells that contributed to the rebuilding of the Treg cell pool. These studies illustrate that infection‐induced loss of IL‐2 and Treg cells is transient, and despite the ability to generate thymically derived Treg cells, those peripheral Treg cells that survive the acute “crash” are preferentially maintained.

## Graphical abstract

Acute *T. gondii* infection caused thymic atrophy, reduced IL‐2, and a sharp decline in Treg cells. As infection resolves, IL‐2 and thymic function recover, which partially restores Tregs. Fate‐mapping shows thymic Treg generation remains intact, but surviving peripheral Tregs chiefly rebuild the pool. Antiparasitic treatment can enhance thymic Treg generation.

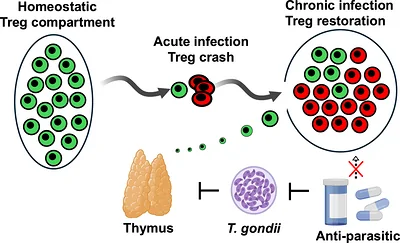

**Abbreviations**
d.p.i: days postinfection
IL‐2/15: interleukin‐2/15
*T. gondii*
: *Toxoplasma gondii*
Treg: regulatory T cell

## 1 Introduction

Thymically derived Foxp3^+^ regulatory T (Treg) cells play a pivotal role in the induction and maintenance of tolerance, and the homeostatic processes that maintain this population of cells are well characterized, as is the ability of Treg cells to limit autoimmunity [1, 2, 3, 4, 5]. The Treg compartment consists of a diverse population of quiescent central Treg (cTreg) cells defined by a naïve‐like phenotype and activated effector (eTreg) Treg cells associated with increased suppressive capacity [6]. There is recognition that Treg cell activity affects the outcome of a variety of infections, but these challenges can also alter Treg cell homeostasis. For example, while some infections (malaria and helminths) lead to increased Treg cell populations [7, 8, 9, 10], some acute systemic infections (*T. gondii, Listeria monocytogenes*, Vaccinia virus) result in a marked restriction of the Treg compartment [11, 12, 13]. During toxoplasmosis, restriction of Treg activity enables the generation of pathogen‐specific effector responses required to control infection [11]. Nevertheless, Treg cell activity during toxoplasmosis is important to prevent immune‐mediated pathology [14, 15]. Several proposed mechanisms contribute to this infection‐induced loss of Treg cells, including the reduced production of the Treg cell growth factor Interleukin‐2 [11, 12, 13], while the ability of Interferon gamma (IFN‐γ) to promote broad immune cell expression of PD‐L1 limits effector PD‐1^+^ Treg cell populations [16]. In addition, during experimental toxoplasmosis, the thymus undergoes a marked involution and decreased thymic output [17], but whether this contributes to the loss of the peripheral pool of Treg cells is unknown.

Infection with *T. gondii* is characterized by an acute phase associated with systemic inflammation, but as the immune system mediates parasite control, the ability to form a latent cyst stage contributes to pathogen persistence and, in certain mouse models, results in chronic inflammation [18, 19]. This provides a nonresolving model of infection, and it is unclear how this might influence Treg cell populations. When these studies were initiated, it was unclear whether the transition from acute to chronic phases of infection results in restoration of IL‐2 production and thymic output or whether the marked restriction of the Treg cell population during acute infection has any long‐term impact on Treg cell homeostasis. The datasets presented here illustrate that the infection‐induced Treg cell crash is followed by restoration of IL‐2 production but a sustained dominance of effector Treg cells. Surprisingly, the marked thymic hypertrophy was not accompanied by a reduction in the ability of this tissue to generate Treg cell populations. To determine whether newly generated or inflammation‐experienced, “survivor” Treg cells contributed to the rebuilding of the Treg pool, we utilized the conditional Treg‐specific fate mapping mouse model (Foxp3^eGFPCre/ERT2^Ai14^CAG‐LSL‐Tdtomato‐Flox/Flox^) and found that in chronically infected mice, the survivor eTreg cells were the dominant contributors to the long‐term Treg cell pool. While antiparasitic drug treatment reduces parasitic replication and inflammation associated with chronic toxoplasmosis, this also resulted in increased generation of thymic Treg cells, but the Treg compartment remained skewed to inflammation‐experienced Treg cells. These datasets indicate that during toxoplasmosis, there is a remarkable capacity to restore the Treg cell compartment, but ongoing inflammation continues to shape the composition of the Treg cell populations.

## 2 Results

**FIGURE 1:**
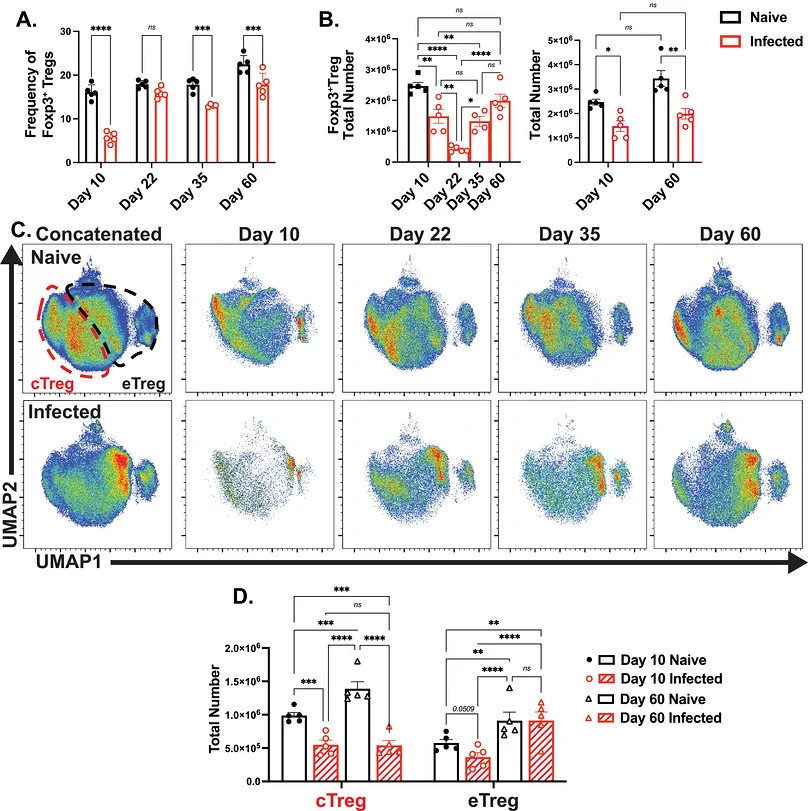
Analysis of Treg cell kinetics during infection. C57BL/6 mice were infected with 20 ME49 cysts by intraperitoneal (I.P.) injection and analyzed at day 10, 22, 35, and day 60, along with age‐matched naïve controls. The Foxp3^+^ Treg compartment was assessed at the indicated time points and analyzed by high‐parameter flow cytometry. (A,B) Quantification of the frequency and total number of splenic Foxp3^+^ Treg cells as determined by flow cytometry at days 10, 22, 35, and 60 for the respective cohorts. *N* = 5 per group. 1 representative experiment shown of 2. Statistical significance determined by two‐way ANOVA and Tukey's multiple correction; (not significant, ns, *p* > 0.05, *p < 0.05, **p < 0.005). Data are presented as x̄ ± SEM. (C). UMAP analysis conducted on splenic Foxp3^+^Treg cells across indicated time points between naïve and infected cohorts. (D). Quantification of the total number of splenic Foxp3^+^CD25^+^Bcl2^+^ cTreg and CD25^−^Bcl2^−^eTreg cells as determined by flow cytometry at day 10 (naïve and infection) and day 60 (naïve and infection). *N* = 5 per group, with 1 representative experiment shown of 2 performed. Statistical significance determined by two‐way ANOVA and Tukey's multiple correction; (not significant, ns, *p* > 0.05, *p < 0.05, **p < 0.005, ****p < 0.0001). Data are presented as x̄ ± SEM.

To investigate the long‐term impact of the infection‐induced restriction of Treg cell populations, cohorts of C57BL/6 mice were infected with *T. gondii* and analyzed at days 10, 22, 35, and 60 to reflect acute and chronic phases of infection. Age‐matched naïve controls were included for each time point. As expected [11, 12, 16], there was a marked reduction in the frequency of splenic Foxp3^+^ Treg cells at 10 days postinfection (d.p.i), and while the frequency of Foxp3^+^Treg cells increased over subsequent time points, the overall frequency of Treg cells was reduced compared with age‐matched naïve controls (Figure 1A). Quantification of the total number of splenic Foxp3^+^ Treg cells illustrates the acute reduction in numbers between days 10 and 22, which was followed by a gradual increase in Treg cell numbers by day 35. However, at 60 d.p.i., there remained a significant reduction in the numbers of Treg cells compared with age‐matched naïve controls (Figure 1B). To visualize the composition and distribution of the Treg cell compartment throughout infection, an antibody panel was utilized to distinguish cTreg vs eTreg cell populations (Figure S1 [16, 20]) and Uniform Manifold Approximation and Projection (UMAP) analysis of splenic Foxp3^+^ Treg cells from naïve and infected C57BL/6 mice at each timepoint was conducted. Concatenated naïve and infected samples across the timepoints analyzed illustrate that infection induces a phenotypic shift in the Treg compartment (Figure 1C, concatenated). Notably, this alteration was observed as early as day 10, and each subsequent time point shows the Treg compartment of infected mice remains distinct from age‐matched naïve controls and is dominated by eTreg cells (Figure 1C). Previous studies have shown that Treg cell numbers increase with age [21, 22, 23], and in agreement, quantification of the splenic cTreg and eTreg cells illustrates an increase in both Treg cell subsets with age in the naïve cohort (Figure 1D). However, within the infected cohort, beginning at day 10, there is a preferential loss of cTreg cells that is maintained through day 60 (Figure 1D). Thus, the early restriction of Treg cells during infection is transient, but chronic infection is associated with a reduced Treg cell pool that is dominated by eTreg cells.

**FIGURE 2:**
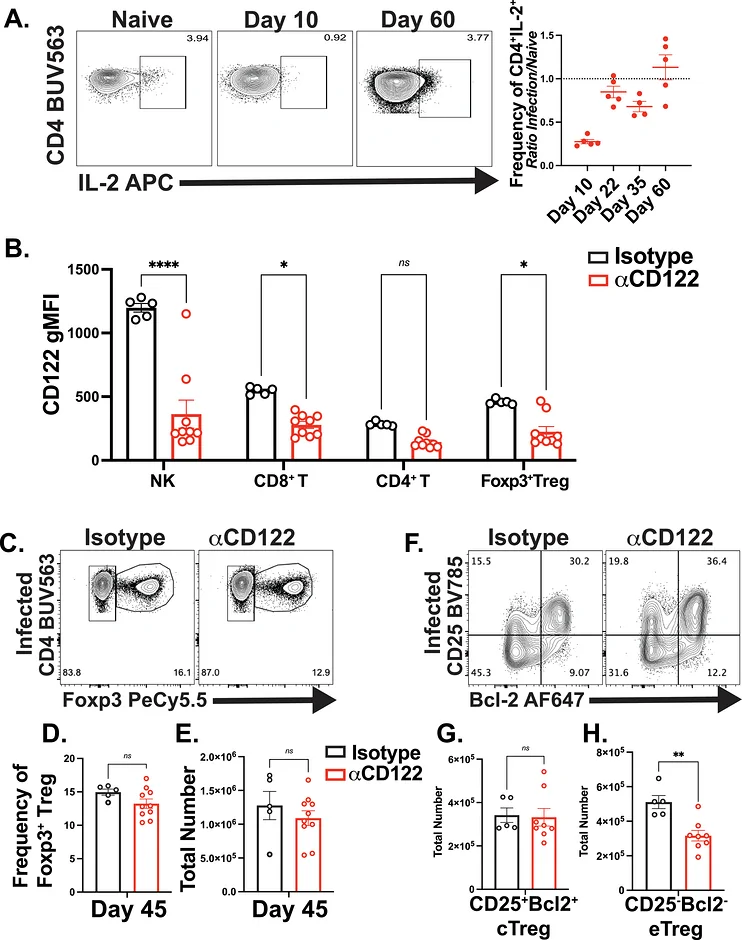
IL‐2R signaling supports eTreg cells during chronic infection (A). C57BL/6 mice were infected I.P. with 20 ME49 cysts and analyzed at days 10, 22, 35, and 60, along with age‐matched naïve controls. Whole splenocytes were stimulated with PMA/Ionomycin, and CD4^+^ T cell production of IL‐2 was assayed by flow cytometry. Representative flow cytometry plots showing IL‐2‐producing CD4^+^ T cells from infected and naïve mice. The ratio was calculated as CD4^+^IL‐2^+^ T cells (infected)/CD4^+^IL‐2^+^ T cells (naïve) at the indicated time points. *N* = 5 per group. 1 representative experiment shown of 2. Data are presented as x̄ ± SEM. (B) C57BL/6 mice infected with 20 ME40 cysts I.P., and treated twice weekly with αCD122 or isotype control beginning at day 35 of infection. Quantification of the gMFI of CD122 on splenic NK, CD8^+^ T, Conventional CD4^+^ T, and Treg cells as determined by flow cytometry. Statistical significance determined by Two‐way ANOVA and Tukey's multiple correction; (not significant, ns, *p* > 0.05, *p < 0.05, ****p < 0.0001). Data are presented as x̄ ± SEM. (C–E) Representative flow plot depicting splenic Foxp3^+^ Treg cells at day 45 postinfection from isotype control and αCD122‐treated mice. Quantification of the frequency and total number of splenic Foxp3^+^ Treg cells as determined by flow cytometry. (F–H). Flow plot depicting splenic Foxp3^+^ central (CD25^+^Bcl2^+^) and effector (CD25^−^Bcl2^−^) Treg cells at day 45 postinfection from isotype control or αCD122‐treated mice. Quantification of the total number of splenic Foxp3^+^ cTreg:eTreg cells as determined by flow cytometry. *N* = 5–10 per group. 1 representative experiment shown of 2. Statistical significance determined by unpaired *T*‐test (not significant, ns, *p* > 0.05, **p < 0.005).

Basal production of IL‐2 by conventional CD4^+^ T cells is critical for Treg cell homeostasis [24], and multiple studies have proposed that the reduced IL‐2 during acute toxoplasmosis contributes to the acute loss of Treg cells [11, 12]. Consistent with previous studies, in naïve mice a small population of IL‐2^+^CD4^+^ T cells was detected, but by 10 d.p.i was markedly reduced (Figure 2A). However, by day 22 and onward, the ability of CD4^+^ T cells to produce IL‐2 was re‐established (Figure 2A). In an effort to assess whether IL‐2 contributed to the restoration and/or maintenance of the Treg cell compartment in chronically infected mice, we opted to use a well‐characterized antibody (clone: TM‐B1 [25]) that blocks CD122, one of the signaling receptor chains of the IL‐2R signaling complex used by IL‐2 and IL‐15 (both cytokines important in Treg cell homeostasis). Therefore, C57BL/6 mice 30 d.p.i were treated with twice weekly injection of αCD122 blocking antibody I.P. and assessed at day 45. To validate this approach, expression of CD122 was quantified on splenic NK, CD8^+^ T, conventional CD4^+^ T, and Foxp3^+^ Treg cells using an αCD122 (TM‐B1) detection antibody. Following CD122 blockade, there was a notable reduction in the ability to detect CD122 expression, especially on NK, CD8^+^ T, and Foxp3^+^Treg cells (Figure 2B). Blockade with αCD122 resulted in reduced frequency and total number of splenic NK cells and CD8^+^ T cells, but had minimal impact on splenic conventional CD4^+^ T cells (Figure S2A–C).

The use of a tetramer to identify *T. gondii*‐specific CD4^+^ and CD8^+^ T cells in the brain illustrates that the overall frequency and number of the parasite‐specific effector response was largely preserved following αCD122 blockade (Figure S2D–F). Additionally, blockade did not impact parasite burden (Figure S2G) and did not result in increased mortality (data not shown). Notably, CD4^+^ and CD8^+^ T cells’ expression of CD122 was comparable between control and αCD122 groups, indicating that the blocking antibody is unlikely to be acting on cells within the brain (Figure S2H). Analysis of the Treg compartment at day 45 showed that αCD122 blockade did not impact these populations in the central nervous system (Figure S2I), and had minimal impact on the splenic Foxp3^+^Treg cell compartment (Figure 2C–E). A refined analysis of cTreg and eTreg subsets revealed that cTreg cells were largely unaltered by frequency and number, but there is a reduction in eTreg cell populations (Figure 2F–H). Together, these data indicate that transient blockade of CD122 has minimal impact on the parasite‐specific effector response and does not impair resistance to the chronic phase of *T. gondii* infection, but the CD122 signaling axis (mediated by IL‐2 or IL‐15) preferentially influences the size and maintenance of eTreg cell populations during this phase of infection.

**FIGURE 3:**
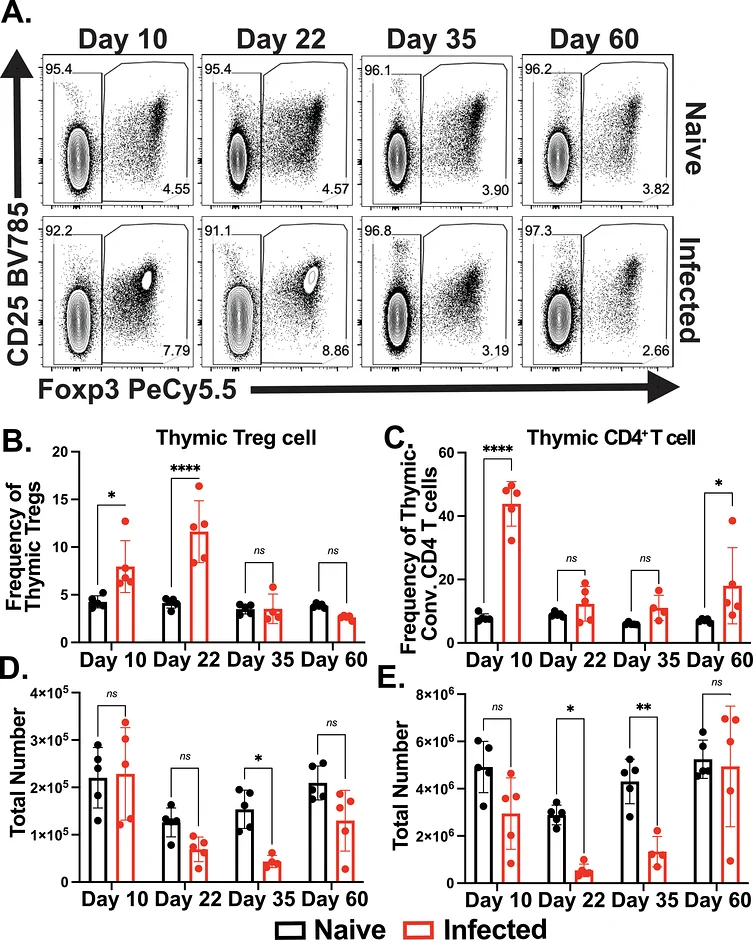
Impact of infection on thymic Treg cells. C57BL/6 mice were infected I.P. with 20 ME49 cysts and analyzed at days 10, 22, 35, and 60, along with age‐matched naïve controls. Thymi were assessed at the indicated timepoints and analyzed by high‐parameter flow cytometry. (A) Flow cytometry plot depicting CD25 expression and Foxp3 expression on single‐positive CD4^+^ thymocytes to identify Foxp3^+^CD25^+^ thymic Treg cells across indicated timepoints. (B–E) Quantification of the frequency and total number of thymic Treg and CD4^+^ T cells at the indicated timepoints. *N* = 5 per group; 1 representative experiment shown of 2. Statistical significance determined by two‐way ANOVA and Tukey's multiple correction (not significant, ns, *p* > 0.05, *p < 0.05, ****p < 0.0001). Data are presented as x̄ ± SEM.

Previous studies have shown that infection with *T. gondii* leads to marked thymic involution with reduced output of newly generated conventional T cells [17]. Since the thymus is the major source of Treg cells, it seemed likely that infection‐induced thymic involution could also reduce production of thymic Treg cells and contribute to the Treg crash. As expected, analysis of thymic T cells at day 10 postinfection revealed a marked loss of thymic cellularity (most notably the CD4^+^CD8^+^ thymocytes—data not shown) and an increased frequency, but not number, of CD4^+^ T and Treg cells compared with naïve controls (Figure 3A–E). Notably, the frequency of CD4^+^Foxp3^+^ thymic Treg cells remained high through day 22 but returned to basal levels during the chronic phase of infection; the total number of Treg cells slightly decreased during the course of infection and followed similar kinetics to conventional CD4^+^ T cells in the thymus (Figure 3A–E). Thus, despite thymic involution during acute infection, the ability to generate thymic Treg cells is largely preserved by frequency and number, but during chronic infection there is a modest defect in thymic output.

The datasets described above suggest that restoration of the Treg cell compartment (Figure 1) could be derived from the restricted pool of survivors or newly derived thymic Treg emigrants. To distinguish between these possibilities, a conditional Treg‐specific fate‐mapping mouse model (Foxp3^eGFPCre/ERT2^Ai14^CAG‐LSL‐Tdtomato‐Flox/Flox^) was utilized. In this system, treatment with tamoxifen enables Cre/ERT2 translocation to the nucleus and excision of the lox‐stop‐lox site to enable constitutive and heritable expression of Tdtomato. In initial studies with these reporter mice, Treg cells were labeled at the peak of the crash (day 10) with a single bolus of 8 mg of Tamoxifen (Figure S3A). This approach resulted in 75%–88% of Treg cells expressing Tdtomato^+^Treg cells (75%–88%) in the Foxp3^eGFPCreERT2^Ai14^Tdtomato+/+^ mice across various tissues (Figure S3B). While we initially chose to label Treg cells at the peak of the Treg crash, the time frame at day 10 coincides with peak inflammation, and high‐dose tamoxifen was associated with toxicity and high mortality (Figure S3C). Therefore, we opted to use two doses [26] of 8 mg Tamoxifen via oral gavage and let the mice rest for ∼14 days [27] to allow the mice to recover from any toxic effects associated with high‐dose tamoxifen treatment [28, 29, 30].

**FIGURE 4:**
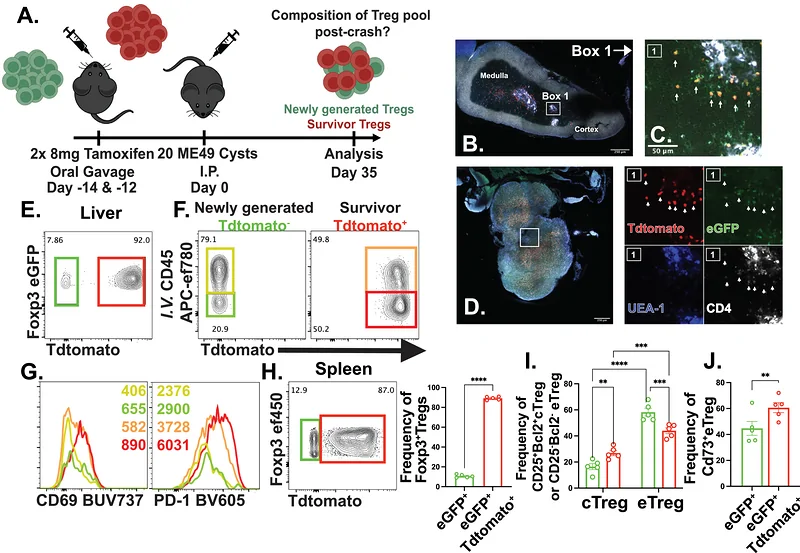
Tracing Treg cells during infection. Foxp3^eGFP Cre/ERt2+/+^Ai14^Tdtomato+/+^ mice were treated with two doses of tamoxifen (8 mg × 2) by oral gavage, rested for ∼2 weeks, and then infected with 20 ME49 cysts by I.P. Naïve and infected mice were euthanized and the Treg compartment assessed at day 35 postinfection. (B, C) Whole C3D‐cleared thymus from naïve and infected Treg reporter mice was harvested, cleared, and analyzed by immunofluorescence microscopy. 1 representative image depicting the naïve thymus. Box 1 is a representative image of a 40X zoom within the medullary region of the naïve thymus. Single‐channel images for each representative marker are displayed below the Box 1 image. (D) 1 representative image depicting the infected thymus at day 35 postinfection. White box is the representative region for the video in Figure S3. (E) Flow plot depicting eGFP^+^ newly generated Treg cells (green box) and eGFP^+^Tdtomato^+^ Survivor Treg cells (red box) harvested from the liver. (F) Intravenous labeling with anti‐CD45 was conducted 3 min before euthanasia to allow discrimination of tissue‐resident (αCD45^−^) and circulating (αCD45^+^) cells. Flow plot depicting tissue‐resident (αCD45^−^) and circulating (αCD45^+^) cells within the eGFP^+^ newly‐generated Treg cells and eGFP^+^Tdtomato^+^ Survivor Treg cells fractions harvested from the liver. (G) Representative histogram shows geometric mean intensity (gMFI) of CD69 and PD‐1 expression on tissue‐resident (αCD45^−^) and circulating (αCD45^+^) cells within the eGFP^+^ newly generated Treg cells and eGFP^+^Tdtomato^+^ Survivor Treg cells fractions harvested from the liver. (H) Flow plot depicting eGFP^+^ newly‐generated Treg cells (green box) and eGFP^+^Tdtomato^+^ Survivor Treg cells (red box) harvested from the spleen. (I) Quantification of the frequency of splenic Foxp3^+^ cTreg:eTreg cells as determined by flow cytometry. *N* = 3–5 per group; 1 representative experiment shown of 3. Statistical significance determined by two‐way ANOVA and Tukey's multiple correction; (**p < 0.005, ***p < 0.001, ****p < 0.0001). Data are presented as x̄ ± SEM. (J) Quantification of the frequency of splenic Foxp3^+^CD73^+^eTreg cells within eGFP^+^ newly‐generated Treg cells and eGFP^+^Tdtomato^+^ Survivor Treg cells as determined by flow cytometry. *N* = 3–5 per group; 1 representative experiment shown of 3. Statistical significance determined by paired *T*‐test; (**p < 0.005). Data are presented as x̄ ± SEM.

To assess the impact of infection on the Treg pool, Foxp3^eGFPCre/ERT2^Ai14^CAG‐LSL‐Tdtomato‐Flox/Flox^ mice were treated twice with tamoxifen, rested for 2 weeks before infection, and then analyzed at 35 dpi (Figure 4A). To confirm that this approach can identify newly generated Treg cells, immunofluorescence imaging of whole C3D‐cleared thymus from naïve Foxp3^eGFPCre/ERT2^Ai14^CAG‐LSL‐Tdtomato‐Flox/Flox^ mice was conducted. In the naïve C3D‐cleared thymus, clusters of eGFP^+^Tdtomato^+^ Treg cells are located toward the center of the thymus, within the medullary region (Figure 4B,C). Box 1 represents a 40X zoom of a region in the medulla to illustrate the dual expression of eGFP^+^ and Tdtomato^+^ in Survivor Tregs, as the expression and visualization of eGFP was dim compared with Tdtomato. While eGFP^+^ Treg cells are less numerous and observed in the outer regions (cortex) of the thymus (Figure 4B; Figure S3D), these locations reflect the presence of mature (medullary) and developing (cortex) thymocytes and indicate that this genetic approach can distinguish new and mature Treg cells within the thymus.

At 35 d.p.i., infected Foxp3^eGFPCre/ERT2^Ai14^CAG‐LSL‐Tdtomato‐Flox/Flox^ mice exhibited an involuted thymus and a z‐stack acquired across 100 µm in the Z‐axis revealed the presence of both eGFP^+^Tdtomato^+^ and eGFP^+^ Treg cells throughout the thymus (Figure 4D; Figure S3E Video), indicating that an involuted thymus with disrupted thymic structure maintains the capacity to generate new thymic Treg cells. Analysis of the newly generated and survivor Treg cells in the periphery (liver and spleen—both sites affected by *T. gondii*) revealed that the majority (>90%) of the Treg pool in the liver was derived from the survivor pool (Figure 4E). Previous studies have found that newly generated T cells can be commonly found within the tissue vasculature, and intravenous labeling with αCD45 confirmed that the eGFP^+^ Treg cells in the liver were preferentially localized in the vasculature (Figure 4F). In contrast, the survivor Treg cells were equally distributed between the vasculature and parenchyma and expressed higher levels of CD69 and PD‐1, two markers associated with activation and tissue residency (Figure 4G). Characterization of the splenic Treg compartment confirmed the dominance of the survivor Treg cells (Figure 4H), while the use of CD25 and Bcl‐2 expression revealed that a portion of the newly generated Treg cells adopted a CD25^low^Bcl2^low^eTreg phenotype (Figure 4I). However, these newly generated eTreg cells expressed reduced levels of CD73, a surface enzyme that negatively regulates adenosine monophosphate metabolism, compared with survivor eTreg cells (Figure 4J). Thus, while the survivor Treg cells dominate the Treg cell pool, a portion of the thymic Treg cells that arise following the collapse do contribute to the eTreg cell population.

**FIGURE 5:**
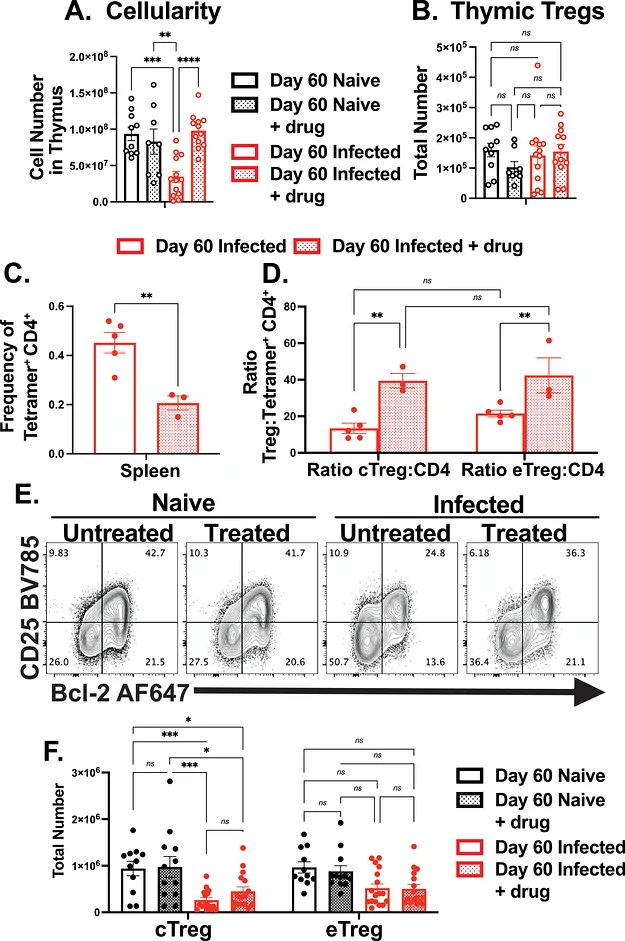
Antiparasitic drug treatment restores thymus cellularity. C57BL/6 mice were infected I.P. with 20 ME49 cysts, and antiparasitic treatment started at day 35. Cohorts of mice assessed at day 60 by high‐parameter flow cytometry. (A) Quantification of the total number of cells harvested from the thymus across conditions. (B) Quantification of the total number of thymic Foxp3^+^ Treg cells across conditions. *N* = 3–5 per group. Pooled data from three independent experiments. Statistical significance determined by one‐way ANOVA; (not significant, ns, *p* > 0.05, *p < 0.05, **p < 0.005, ***p < 0.001, ****p < 0.0001). (C) Quantification of the frequency of parasite‐specific Tetramer^+^ CD4^+^ T cells in the spleen across conditions. *N* = 3–5 per group. 1 representative experiment shown of 3. Statistical significance determined by one‐way ANOVA; (not significant, ns, *p* > 0.05, *p < 0.05, **p < 0.005, ***p < 0.001, ****p < 0.0001). (D) The ratio of c:eTreg cells to parasite‐specific CD4^+^ T cells based on total number as determined by flow cytometry. *N* = 3–5 per group. 1 representative experiment shown of 3. Statistical significance determined by two‐way ANOVA; (not significant, ns, *p* > 0.05, *p < 0.05, **p < 0.005, ***p < 0.001, ****p < 0.0001). (E,F) Flow plot depicting splenic CD25^+^Bcl2^+^ cTreg and CD25^−^Bcl2^−^ eTreg cells across naïve and infected cohorts and treatment groups. Quantification of the total number of cTreg:eTreg cells as determined by flow cytometry. *N* = 3–5 per group. Pooled data from three independent experiments. Statistical significance determined by two‐way ANOVA; (not significant, ns, *p* > 0.05, *p < 0.05, **p < 0.005, ***p < 0.001, ****p < 0.0001).

One feature of the chronic stage of *T. gondii* infection is the presence of low levels of parasite replication and ongoing inflammation in the CNS, muscle, and lungs [31, 32] that could contribute to the maintenance of the eTreg phenotype. To determine whether the underlying parasite replication and low‐grade inflammation impact developing Treg cells in the thymus or contribute to the Treg cell phenotypes in peripheral tissues, C57BL/6 mice were treated with sulfadiazine and pyrimethamine beginning at 35 d.p.i. to limit parasite replication and ameliorate inflammation [33, 34, 35], and assessed at 60 d.p.i. This treatment restored thymic cellularity (Figure 5A) but did not alter thymic Treg cell numbers (Figure 5B). As expected, treatment led to a reduction in the frequency of parasite‐specific CD4^+^ T cells (Figure 5C) and an increase in the ratio of Treg:Parasite‐specific T cells (Figure 5D). Additional analysis of the peripheral Treg pool in the spleen illustrated that antiparasitic drug treatment increased the frequency of cTreg cells but did not fully restore the peripheral cTreg pool (Figure 5E,F). Thus, the ability of antiparasitic drugs to dampen inflammation and reduce the parasite‐specific effector response did not fully restore the Treg cell response to homeostatic levels.

**FIGURE 6:**
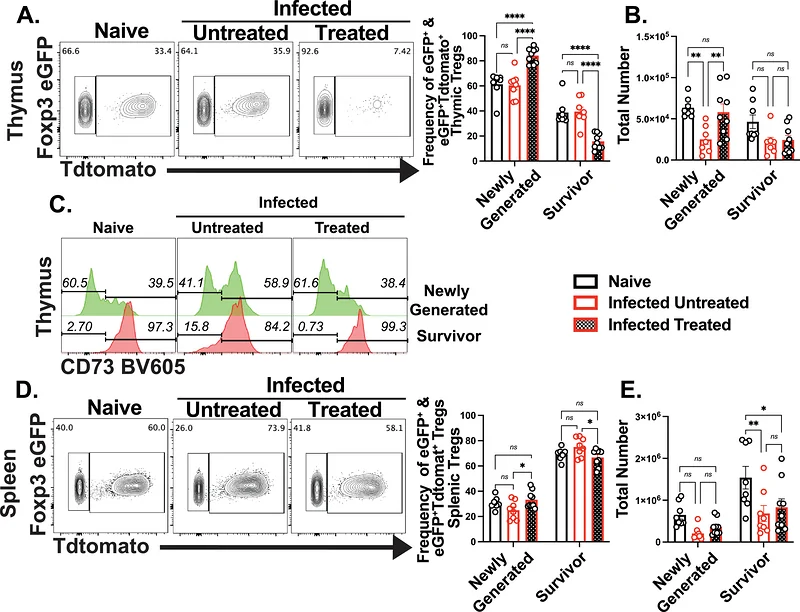
Impact of antiparasite drug treatment on Treg cell pool. Foxp3^eGFP Cre/ERt2+/+^Ai14^Tdtomato+/+^ mice were treated with two doses of tamoxifen (8 mg × 2) by oral gavage, rested for ∼2 weeks, and then infected with 20 ME49 cysts by I.P. Mice began antiparasitic treatment at day 35, administered via drinking water, and the Treg compartment assessed at day 60 by high‐parameter flow cytometry. (A,B) Flow plot depicting eGFP^+^ newly‐generated Treg cells and eGFP^+^Tdtomato^+^ Survivor Treg cells harvested from the thymus. Quantification of the frequency and total number of thymic eGFP^+^ newly‐generated Treg cells and eGFP^+^Tdtomato^+^ Survivor Treg cells as determined by flow cytometry. (C) 1 representative histogram depicting CD73 expression on eGFP^+^ newly‐generated Treg cells and eGFP^+^Tdtomato^+^ Survivor Treg cells in the thymus. (D,E) Flow plot depicting eGFP^+^ newly‐generated Treg cells and eGFP^+^Tdtomato^+^ Survivor Treg cells harvested from the spleen. Quantification of the frequency and total number of splenic eGFP^+^ newly‐generated Treg cells and eGFP^+^Tdtomato^+^ Survivor Treg cells as determined by flow cytometry.

**FIGURE 7:**
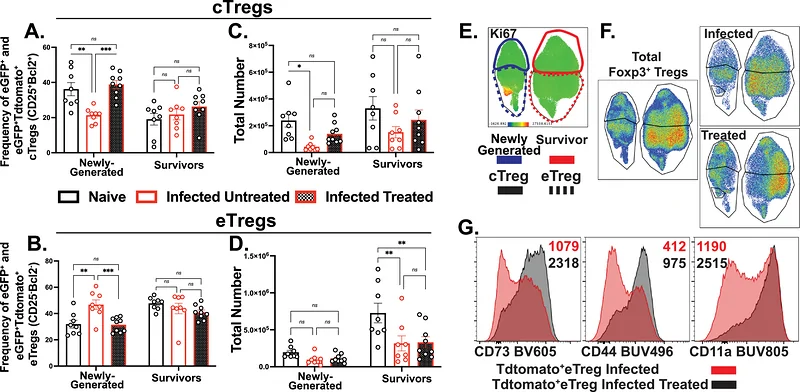
Antiparasite drug treatment fails to restore inflammation‐experienced eTreg numbers. Foxp3^eGFP Cre/ERt2+/+^Ai14^Tdtomato+/+^ mice were treated with two doses of tamoxifen (8 mg × 2) by oral gavage, rested for ∼2 weeks, and then infected with 20 ME49 cysts by I.P. Mice began antiparasitic treatment at day 35, administered via drinking water, and the Treg compartment assessed at day 60 by high‐parameter flow cytometry. (A–D) Quantification of the frequency and total number of splenic eGFP^+^ newly‐generated cTreg:eTreg and eGFP^+^Tdtomato^+^ Survivor cTreg:eTreg cells as determined by flow cytometry. *N* = −3–5 per group. Pooled data from two independent experiments. Statistical significance determined by two‐way ANOVA and Tukey's multiple correction; (not significant, ns, *p* > 0.05,*p < 0.05, **p < 0.005, ***p < 0.001, ****p < 0.0001). Data are presented as x̄ ± SEM. (E,F) UMAP visualization of Foxp3^+^ Treg cells from Foxp3^eGFP Cre/ERt2+/+^Ai14^Tdtomato+/+^ mice, and visualization of Ki67 expression is overlaid onto UMAP. UMAP visualization of Foxp3^+^ Treg cells from infected (untreated) and antiparasitic drug‐treated cohorts. (G) Representative histogram depicting geometric mean intensity of CD73, CD44, and CD11a expression on splenic Tdtomato^+^Survivor eTreg cells.

Next, the use of the Treg tracing model and antiparasitic drug treatment was combined to assess if decreased inflammation differentially impacted newly generated or survivor Treg cell populations. Cohorts of Foxp3^eGFPCre/ERT2^Ai14^CAG‐LSL‐Tdtomato‐Flox/Flox^ mice were treated with tamoxifen, infected two weeks later, and at day 35 administered antiparasitic drugs and analyzed at 60 dpi. At this late time point, this treatment resulted in a marked reduction in the frequency of surviving Treg cells within the thymus and an increase in the frequency and number of newly generated Treg cells (Figure 6A,B). When expression of CD73 was used to distinguish developing (CD73^−^) and mature, recirculating (CD73^+^) Treg cells [36] present in the thymus, it revealed that a proportion of newly generated Treg cells upregulate CD73 and thus have matured and returned to the thymus. However, newly generated CD73^+^ Treg cells were reduced upon treatment with antiparasitic drugs (Figure 6C). Thus, low levels of ongoing parasite replication and associated inflammation contribute to systemic thymic involution during *T. gondii* infection, which reduces the emergence of newly generated thymic Treg cells. However, in the spleen, this treatment only led to a subtle reduction in the frequency of survivor Treg cells (Figure 6D), and when compared with naïve mice, did not improve Treg cell numbers across newly‐generated and survivor Treg populations (Figure 6E). Evaluation of the cTreg:eTreg frequency showed that antiparasitic treatment did result in a change in the ratio of newly‐generated cTreg:eTreg cells (Figure 7A,B). This analysis illustrates that antiparasitic treatment results in a partial restoration of newly generated cTreg cells and emphasizes that the survivor eTreg cell compartment is numerically reduced but is not restored (Figure 7C,D). Phenotypic analysis and UMAP visualization illustrate expression of Ki67, a marker of cell proliferation, solely in the newly generated Treg compartment (Figure 7E) and a notable phenotypic shift in the survivor Treg compartment upon antiparasitic treatment (Figure 7F). While there was no restoration in the numbers of the survivor Treg compartment, expression of markers associated with Treg cell activity (CD73, CD44, CD11a) were found to be upregulated on survivor eTreg cells following antiparasitic treatment (Figure 7G). Thus, the ability of the antiparasitic drug treatment to inhibit parasitic replication and remove inflammation can alter Treg cell activation states, but does not fully restore peripheral Treg cell populations to preinfection levels. These datasets indicate that the initial Treg “crash” that restricts and alters Treg cell populations during the early stages of infection has a long‐lasting impact on the size of the peripheral Treg pool that re‐builds the niche and that chronic infection‐induced inflammation continues to shape the Treg cell populations.

## 3 Discussion

The activity of regulatory T cells during infection is complex and context‐dependent. For example, in murine models of malaria and certain helminth infections, these organisms co‐opt Treg cell activity as a mechanism of immune evasion and pathogen persistence [7, 8, 9, 34, 35, 36, 37, 38]. In other systems, Treg cell activity is critical to limit aberrant immunopathology, and yet the crash of Treg cells appears to be a host mechanism that permits the rapid generation of the effector response [11, 12, 39]. In the context of toxoplasmosis, the numerical restriction but activation of Treg cells allows the development of a parasite‐specific effector response and preserves sufficient Treg cell activity to limit immune‐mediated pathology. While the long‐term consequences of the initial contraction of the Treg cell compartment were unclear, the studies presented here illustrate that the Treg cell collapse associated with *T. gondii* infection is transient and restoration of the Treg cell compartment is associated with the expansion and maintenance of a population of inflammation‐experienced eTreg cells.

There are two main processes associated with acute toxoplasmosis that are likely to affect the rebuilding of Treg cell populations: loss of IL‐2 and thymic involution. Here, we show the early loss of IL‐2 associated with the Treg cell collapse is transient, and despite marked changes in thymic structure and output, the production of thymic Treg cells remained largely intact throughout infection. Since auto‐reactive CD4^+^ T cells are considered a major source of homeostatic IL‐2 [40, 41], this creates a complex immunological circuit in which auto‐reactive T cells support the Treg cell populations that limit their auto‐reactivity. There are few studies that understand the processes that regulate this homeostatic source of IL‐2 [40, 41], and their transient loss and restoration during toxoplasmosis raise new questions about the factors that regulate these events. The cytokines IL‐12 and IL‐27 (both produced and active during *T. gondii* infection) can suppress T cell production of IL‐2 [42, 43, 44, 45], and it is interesting that these cytokines also act on and support Treg cell populations [20, 46, 47, 48, 49, 50]. However, our studies do not readily distinguish whether there are active mechanisms to suppress the production of IL‐2 by these auto‐reactive CD4^+^ T cells, or whether these inflammatory events cause their transient loss but eventual replacement by new auto‐reactive CD4^+^ T cells. Regardless, this infectious model provides a system where some of these broader questions can be addressed to understand how to manipulate the immune system to restore Treg cell homeostasis.

The use of CD122 blockade during chronic infection reduced the eTreg cell population; however, these data sets need to be interpreted with care, since this treatment also impacted the peripheral NK and CD8^+^ T cell populations. As NK cells play a critical role in acute resistance to *T. gondii* and CD8^+^ T cells are required for resistance during the chronic phase, one concern was that this treatment might suppress the parasite‐specific effector responses and increase susceptibility to toxoplasmic encephalitis. While we observed reduced NK cell responses in the periphery, there was a preservation of parasite‐specific CD8^+^ T cells within the CNS and no changes in parasite burden or susceptibility to infection. These data sets are consistent with previous studies that used IL‐2Rα‐depleting antibodies in the setting of a chronic *T. gondii* infection and found no impact on resistance to chronic infection [25]. It is possible that the reduction of Treg cells observed could be a secondary consequence of the marked effects of this blockade on NK cells. However, there is evidence that NK cell production of IFN‐γ acts to negatively regulate Treg cells [51]; therefore, the reduction in eTreg cells following aCD122 blockade is unlikely due to an indirect effect of NK cell depletion. Taken together, these datasets suggest a direct role for CD122 signaling to promote and maintain eTreg homeostasis. Although IL‐2 and IL‐15 both utilize CD122, their effects on T cell development and function are distinct [52], and it is unclear how these cytokines influence different Treg cell subsets in the setting of chronic infection. Because the eTreg cells downregulate the high‐affinity IL‐2Rα (CD25) subunit, it suggests that they are less reliant on IL‐2, and therefore we favor a model in which the eTreg cell population depends upon the ability to compete for IL‐15. Reports that IL‐27 can also promote generation of eTreg cells and improve their responsiveness to IL‐15 by increasing expression of CD122 highlight another potential co‐factor relevant to chronic toxoplasmosis [53] that may contribute to these events.

There has been a long‐standing interest in understanding how inflammatory processes affect Treg cell populations, which is exemplified by studies that showed that exposure to inflammation confers Treg cell preference for nonlymphoid tissues [54]. Similarly, with the realization that Treg cells have the capacity to adopt Th1, Th2, and Th17‐like phenotypes in response to local inflammatory cues [55], it has become clear that Treg cell plasticity underlies the ability to tailor Treg cell activities to distinct inflammatory cassettes associated with different classes of pathogens. However, it has been unclear whether these processes have long‐term effects on Treg cell populations. Indeed, in a model of autoimmune‐mediated inflammation associated with Treg cell activation, epigenetic analysis suggested that this activation is transient and that Treg cells lack the extensive chromatin modifications associated with reactivation and the formation of immunological memory [56]. In that context, we initially expected that in the setting of chronic infection, the ability to limit parasite replication and dampen inflammation would lead to the homeostatic replacement of the surviving Treg cells by newly generated Treg cells. The use of Treg fate‐mapping mice showed that the inflammation‐experienced eTreg cells are not readily replaced by newly generated cTreg cells, which indicates that inflammation‐experienced eTreg cells appear better equipped to persist or have a competitive advantage over newly generated Treg cells. Nevertheless, while previous studies suggested that inflammation‐experienced Treg cells largely lacked functional “memory,” those authors suggested it was possible that chronic exposure to activation signals might imprint a more stable hypersuppressive state. Future studies are needed to determine the basis for the enhanced fitness of these inflammation‐experienced eTreg cells. It will also be important to determine whether inflammation associated with *T. gondii* imprints long‐lasting effects on Treg cell populations, such as heritable chromatin modifications or alterations in the TCR repertoire, and whether this long‐term impact is also observed in additional chronic disease states.

## 4 Materials and Methods

### 4.1 In Vivo Procedures

All mice were housed in the University of Pennsylvania School of Veterinary Medicine vivarium with 12 h light and dark cycles, maintained at a temperature of 68–77°F and humidity of 35%–55% in accordance with institutional guidelines. For all strains of mice used in this study, both males and females were used between 8 and 16 weeks of age, unless otherwise stated. C57BL/6 mice were purchased from Jackson Laboratories at 6–8 weeks of age and housed for 2–4 weeks until used. Foxp3^eGFPCre/ERT2^ (stock: 016961, RRID:IMSR_JAX:016961) and Ai14^CAG‐LSL‐Tdtomato^ (stock: 007908, RRID:IMSR_JAX:007908) were obtained from Jackson Laboratories and crossed to generate Foxp3^eGFPCre/ERT2+/+^Ai14^CAG‐LSL‐Tdtomato+/+^ mice in‐house. Conditional labeling of Treg cells in Foxp3^eGFPCre/ERT2+/+^Ai14^CAG‐LSL‐Tdtomato+/+^ was conducted by oral gavage with two doses of tamoxifen dissolved in corn oil (200 µL @ 40 mg/mL). Ethical oversight of all animal use in this study was approved by the University of Pennsylvania Institutional Animal Care and Use Committee. Mice were infected with ME49, a type II *T*. *gondii* strain, as previously described [16, 56]. Briefly, infections were performed by intraperitoneal injection using 20 cysts of the ME49 strain of *T*. *gondii* that were collected from neural tissue of chronically infected CBA/ca mice.

### 4.2 Tissue Preparation

Single‐cell suspensions were prepared from thymus, spleen, and liver samples for flow cytometry analysis. Thymus and spleens were mechanically processed and passed through a 70 µm nylon filter and then lysed in 1 ml of 0.846% solution of NH_4_Cl for red blood cell lysis. The cells were then washed and stored on ice. For liver preparations, the left renal artery was severed, and the mice were perfused using 10 mL of 1× DPBS. The gallbladder was removed, and the lobes of the liver were mechanically processed over a 70 µm nylon filter and washed. The single‐cell preparations were then resuspended in 20 mL of 37.5% Percoll and centrifuged at 500 g for 20 min at room temperature. The pellet was then resuspended in NH_4_Cl solution for red blood cell lysis, and the cells were washed and stored on ice.

### 4.3 Flow Cytometric Staining and Analysis

Aliquots consisting of 5–8 × 10^6^ cells were washed with ice‐cold 1× DPBS in a 96‐well round‐bottom plate, then incubated in 50 µL volume of viability stain reconstituted in 1× DPBS for 20 min on ice and then washed in 0.2% FACS buffer. The cells were then incubated in 50 µL of Fc block for 30 min on ice, and if identifying for parasite‐specific T cell response, Tetramer‐MHC‐I & Tetramer‐MHC‐II (APC, PE; clone: MHC‐I—SVLAFRRL, MHC‐II—AVEIHRPVPGTAPPS, NIH Tetramer Core) was included during blocking. The cells were washed in 0.2% FACS buffer and then incubated for 20 min on ice in 50 µL of antibody cocktail composed of surface‐stain antibodies in 0.2% FACS buffer supplemented with brilliant stain buffer. The following antibodies were surface stained: CD11a (BUV805; clone: 2D7; RRID:AB_2871232), CD25 (BV785; clone: PC61; RRID:AB_2564131), CD31 (BV711; clone: 390; RRID: AB_2860594), CD44 (BUV496, BV605, AF700, PE, PE‐Cy7; clone: IM7; RRID: AB_2925331, RRID: AB_2274702, RRID: AB_494011, RRID: AB_465664, RRID: AB_469623), CD62L (BV605, BV711, PE‐Cy7; clone: MEL‐14; RRID: AB_2563058, RRID: AB_2564215, RRID: AB_313103), CD69 (BUV737; clone: H1.2F3; NA), CD122 (PE‐Cy5; clone: TM‐b1; RRID: AB_2715962), CD304/Neuropilin‐1 (BB700; clone: V46‐1954; AB_2917459), CXCR3 (BV650; clone: CXCR3‐173; RRID: AB_2563160), GITR (PE‐Cy7; clone: DTA‐1; RRID: AB_647252), ICOS (APC‐Fire750; clone: C398.4A; RRID: AB_2632923), KLRG1 (BV711, FITC, BB630; clone: 2F1; RRID: AB_2629721, RRID: AB_1311265, N/A), PD‐1 (BV421, BV605; clone: 29F.1A12; RRID:AB_2562568, RRID:AB_2562616). The cells were washed in 0.2% FACS buffer and resuspended in 50 µl Foxp3 Perm‐fix cocktail (00‐5523‐00, Thermo Fisher Scientific) for 30 min‐overnight at 4°C. The cells were then washed twice in 1× permeabilization buffer and resuspended in an intracellular staining cocktail composed of intracellular‐stain antibodies diluted in 1× permeabilization buffer for 2 h‐overnight at 4 °C. The following antibodies were intracellular stained: BCL‐2 (AF647; clone: BCL/10C4; RRID: AB_2274702), CD3 (APC‐ef780, BUV395, BUV737, APC, PE, PerCP‐Cy5.5; clone; 17A2, 145‐2C11; RRID:AB_1272181, RRID: AB_2738278, RRID:AB_2738781, RRID: AB_469315, RRID: AB_465496, RRID: AB_893318), CD4 (BUV496, BUV563; clone: GK1.5; RRID:AB_2813886, RRID:AB_2813886), CD8a (BUV563, BUV615, BV650; clone: 53–6.7; RRID:AB_2872946, RRID:AB_2870272, RRID:AB_2563056), cMyc (PE; clone: 9E10: RRID: N/A: catalog #: NB600‐302PE), CTLA‐4 (APC‐R700; clone: UC10‐4F10‐11; RRID:AB_2739350), FOXP3 (PE‐Cy5.5; clone: FJK‐16s; RRID: AB_11218094), HELIOS (PE‐Dazzle594; clone: 22F6; RRID: AB_2565797), Ki67 (BUV395, BV421, AF488, AF700; clone: B56; RRID: AB_2738577, RRID: AB_2686897, N/A, RRID: AB_11204087), NK1.1 (BUV395; clone: PK136; RRID:AB_2738618), NKp46 (BV605, PE‐Dazzle594; clone: 29A1.4; RRID: AB_2562452, RRID: AB_2616666), Tbet (PE‐Cy5; clone: 4B10; RRID: AB_2815071). The cells were then washed twice with 1× permeabilization buffer and then resuspended in 500 µL 0.2% FACS buffer for flow cytometric analysis on a BD Symphony A3 or A5. UMAP analysis was performed using the UMAP plug‐in using the Euclidean distance function with a nearest neighbor score of 15 and a minimum distance rating of 0.5 (v.1802.03426, 2018, 2017, Leland McInness) for FlowJo (v.10.8.1). All stained parameters were included in UMAP analysis except for: Forward & side‐scatter channels, Live/Dead (gated out), and any pregated lineage defining markers, such as CD3, CD4, CD8, when applicable.

### 4.4 In Vitro Cytokine Stimulation

To detect T cell production of cytokines, cells were resuspended in a stimulation cocktail of 0.05 ng·mL^−1^ PMA (Sigma), 0.5 ng·mL^−1^ ionomycin (Sigma), 5 ng·mL^−1^ BFA (BioLegend) and 0.75 µL·mL^−1^ Golgi‐stop (BD Biosciences, 554724) in cRPMI for 3 h at 37°C and 5% CO_2_. Cells were then washed, surface‐stained, and permeabilized as described above for T cell staining. The cytokine‐stain‐prepped cells were then intracellularly stained with a cytokine detection panel for 2 h on ice. The cells were washed and resuspended in 200 µL 0.2% FACS buffer for analysis.

### 4.5 In Vivo Antibody Blockade

Inhibition of IL‐2R signaling was performed beginning at day 30 postinfection by twice‐weekly I.P. injection of αCD122 (clone: TM‐B1, BioXcell) at 200 µg per dose, or rat IgG2b (clone LTF‐2, BioXcell) isotype being used in control groups, and treated cohorts analyzed at indicated time points.

### 4.6 Immunofluorescence Microscopy

Naïve and infected Foxp3^eGFPCre/ERT2+/+^Ai14^CAG‐LSL‐Tdtomato+/+^ mice were euthanized, the thymus was harvested and C3D‐cleared or embedded in Tissue Tek Optimal Cutting Temperature media, and snap frozen in liquid nitrogen. 16 µm sections were cut on a CM3050 S cryostat (Leica) and placed onto charged microscope slides. Following sectioning, slides were rehydrated and fixed in 4% paraformaldehyde for 15 min at RT, then washed 3× with PBS‐T (0.05% Tween‐20). Following fixation, sections were permeabilized with 1% Triton X‐100 (in PBS) for 10 min at RT and washed 3× with PBS‐T. Sections were subsequently blocked with a blocking buffer (containing 5% bovine serum albumin and relevant IgG isotypes) for 10 min at RT and then washed 3× with PBS‐T. Slides and C3D‐cleared tissue were incubated with primary antibody overnight; CD4 BV570 (clone: RM4‐5; RRID: AB_2563051), UEA‐1 DyLight649 (Vector Laboratories: catalog #DL‐1068‐1) at 4°C, washed the following day 5× with PBS‐T, and then mounted using VECTASHIELD Antifade Mounting Medium. Slides and C3D‐cleared tissue were imaged on a Stellaris Falcon (Leica) microscope, and images were processed and analyzed using the Fiji software package (V:2.1.0).

### 4.7 Antiparasitic Drug Treatment

Infected mice were treated with the antiparasitic drugs sulfadiazine (Sigma‐Aldrich, S8626‐25G) and pyrimethamine (Sigma‐Aldrich, SML3579) via drinking water. Sulfadiazine was reconstituted in dimethylsulfoxide (DSMO) to 50 mg/mL and added to drinking water at a final concentration of 250 mg/L. Pyrimethamine was reconstituted in DMSO to 5 mg/mL and added to drinking water at a final concentration of 50 mg/L. Antiparasitic drug‐treated water was administered starting at 35 d.p.i. and refreshed every 3 days for 4 weeks [34, 35].

### 4.8 Quantification of Parasite Burden

100 mg section of the brain was purified for total DNA using the DNeasy Isolation kit (QIAGEN). DNA concentration was assessed using a NanoDrop spectrophotometer and normalized to equal concentration for qPCR analysis (50 ng·µL^−1^). qPCR for parasite burden was conducted using *Toxoplasma*‐specific primers: (forward) 5ʹ‐TCCCCTCTGCTGGCGAAAAGT‐3ʹ and (reverse) 5ʹ‐AGCGTTCGTGGTCAACTATCGATTG‐3ʹ and Power SYBR Green master mix (Applied Biosystems). The qPCR condition settings were a hold phase (occurs only once at the start) for 2 min at 50°C and 10 min at 95°C. The PCR phase (occurred 50×) was 15 s at 95°C and 1 min at 60 °C.

### 4.9 Statistics

Statistical analysis was performed using Prism 10. For comparison of means between two groups, a two‐tailed unpaired Student's *t*‐test was utilized with a 95% CI. Analysis for univariate statistics comparing multiple means was performed using a one‐way ANOVA (family‐wise significance and 95% CI), with post hoc analysis consisting of Fisher's LSD test for direct comparison of two means within the ANOVA, Tukey's multiple comparisons test for comparisons of all means within the test group for multiple‐comparison correction, or Dunn's multiple comparison test for comparisons of every mean to a control mean. For multigroup multivariate analysis, we used a two‐way ANOVA with post hoc analysis, utilizing Sidak's multiple comparisons test for comparisons across two groups with two variables or Tukey's multiple comparisons test for comparisons across multiple groups for multiple variables (also with a 95% CI). *p*‐values <0.05 were considered statistically significant. All error bars in the figures indicate SEM.

### 4.10 Data Limitations and Perspectives

The study has several limitations that need to be addressed. First, mixed cohorts of male and female mice were used in the experiments shown. While mice were age‐matched, the use of both male and female mice limits the interpretation of sex‐related differences that may underlie variability observed within our datasets. Second, we acknowledge that the experimental scheme in Figure 4A suggests 100% labeling efficiency. Unfortunately, we do not have direct readouts of labeling efficiency from these experiments, but given the high degree of labeling in preliminary studies, we believe we are achieving high labeling efficiency with this approach. Finally, the use of a conditional fluorescent reporter line to label and track Treg population dynamics over time is limited by the immunogenicity associated with the fluorescent protein and the potential for background immunogenicity as a confounding factor in population turnover. Despite these limitations, our results indicate that infection‐mediated inflammation dynamically shapes and has long‐lasting effects on Treg cell populations.

## Author Contributions

**Zachary R. Lanzar, Christopher A. Hunter**: conceptualizations. **Zachary R. Lanzar, Christopher A. Hunter**: methodology. **Zachary R. Lanzar**, **Daniel L. Aldridge, Julia N. Eberhard, Elisa Cruz‐Morales**, **Isabella O. Conway**, **Molly E. Bunkofske, Joseph A. Pereira**: investigation. **Melissa Parker**, **Chryssa Kanellopoulou**: resources. **Zachary R. Lanzar, Christopher A. Hunter**: writing – original draft. **Zachary R. Lanzar, Christopher A. Hunter**: review and editing. **Zachary R. Lanzar**, **Christopher A. Hunter**: funding acquisition. **Christopher A. Hunter**: project administration and supervision

## Ethics Statement

All animal experiments were approved by the Institutional Animal Care and Use Committee (IACUC) of the University of Pennsylvania under protocol number 805432 and were conducted in accordance with the U.S. Animal Welfare Act and National Institutes of Health guidelines.

## Conflicts of Interest

Melissa Parker and Chryssa Kanellopoulou are current employees and shareholders of Incyte Corporation. The remaining authors declare no conflicts of interest.

## Supporting information




**Supporting File 1**: eji70265‐sup‐0001‐SuppMat.pdf.

**Supporting File 2**: eji70265‐sup‐0002‐Video 1_Crop.avi.

## Data Availability

The data that support the findings of this study are available from the corresponding author upon reasonable request.
