# Supplementary material for: Impact of Chronic Infection‐Induced Inflammation on TREG Cell Homeostasis
Source: Eur J Immunol. 2026 Aug 20;56(8):e70265. doi: 10.1002/eji.70265 (PMC13490973; doi:10.1002/eji.70265)
Supplement: Supplementary file 1 [file EJI-56-e70265-s001.pdf]

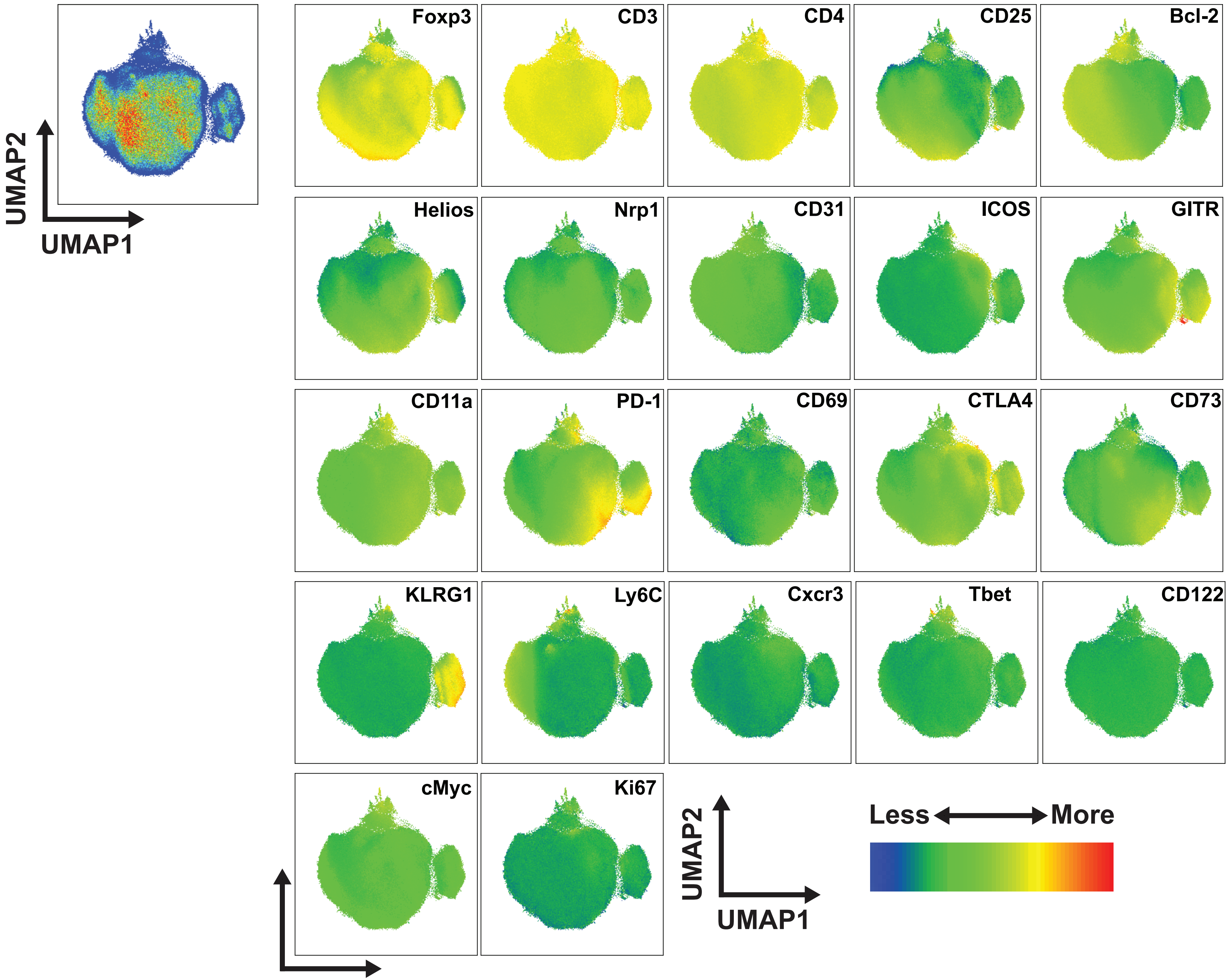

Supplemental Figure 1

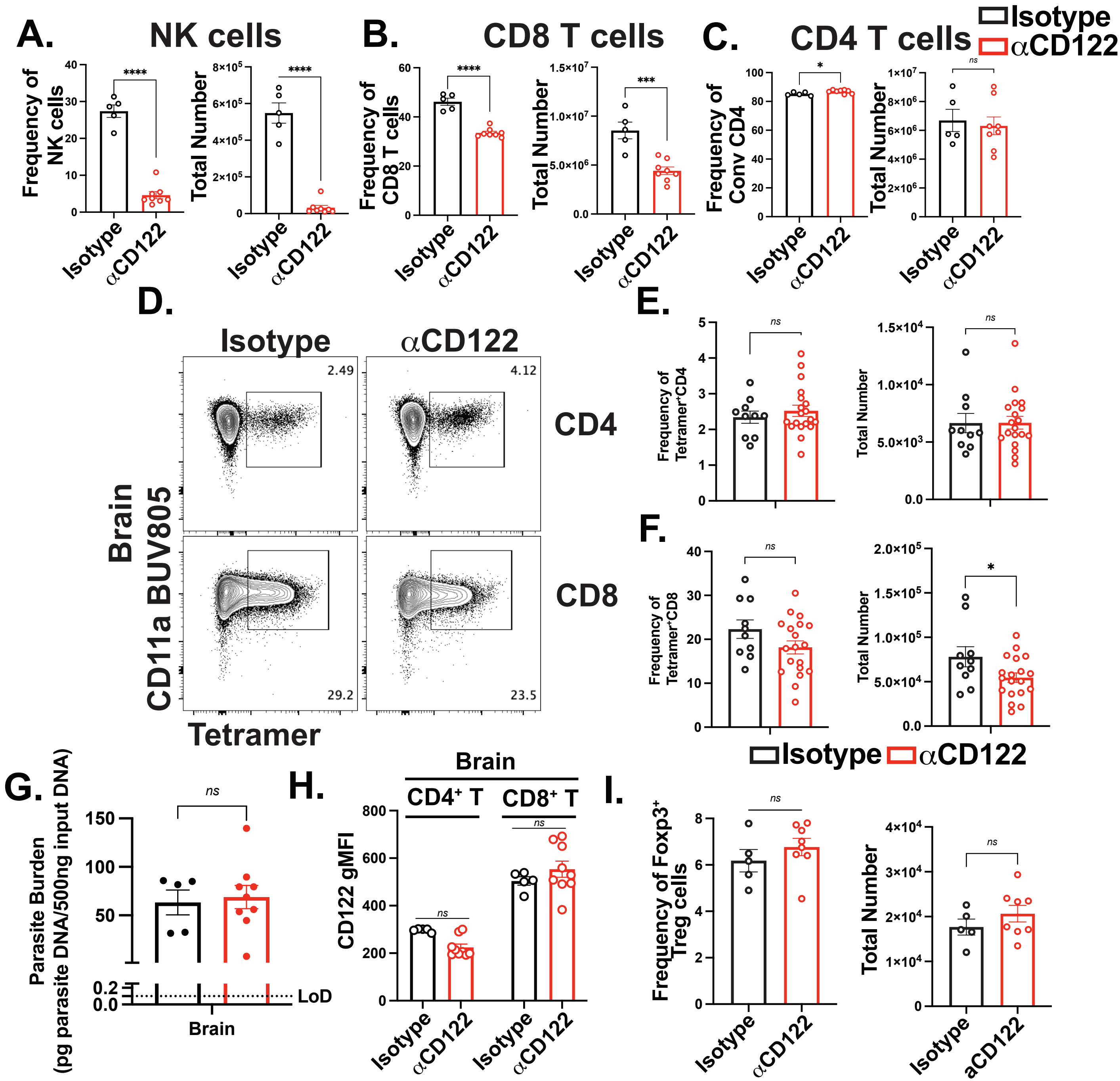

Supplemental Figure 2.

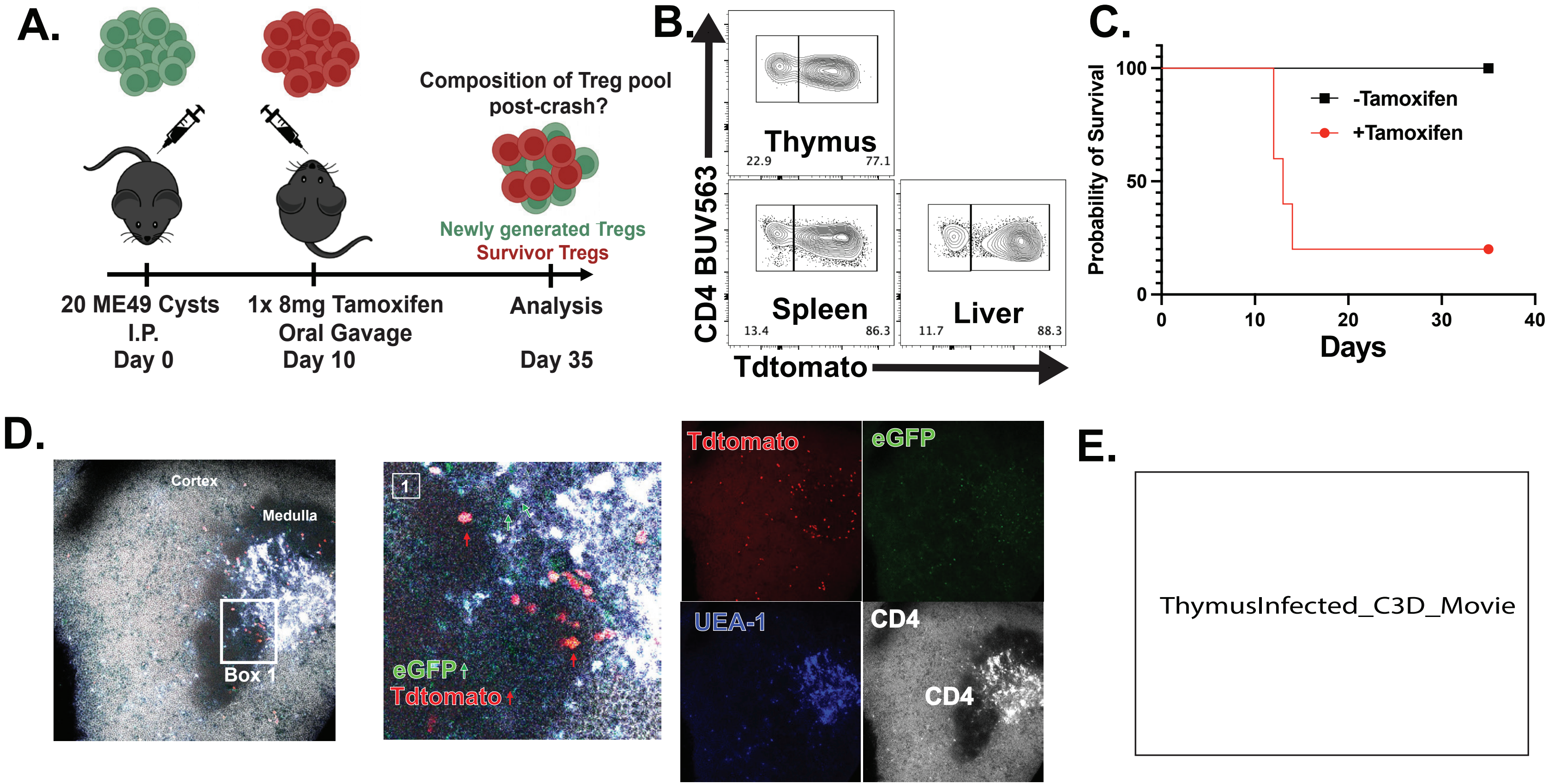

Supplemental Figure 3.

Supplemental Figure Legends:

Supplemental Figure 1: UMAP phenotyping. UMAP visualization of Foxp3$^+$Treg cells harvested from the spleen at days 10, 22, 35, and day 60 from infected and age-matched naïve controls. 22 parameters utilized for UMAP generation.

Supplemental Figure 2: Parasite-specific T cell response is preserved in the brain during αCD122 blockade. C57BL/6 mice infected with 20 ME40 cysts I.P., and treated twice weekly with αCD122 or isotype beginning at day 35 of infection. (A). Quantification of the frequency and total number of splenic NK1.1$^+$ NK cells as determined by flow cytometry. (B) Quantification of the frequency and total number of splenic CD8$^+$ T cells as determined by flow cytometry. (C) Quantification of the frequency and total number of splenic CD4$^+$ T cells as determined by flow cytometry. N=5-10 per group. 1 representative experiment shown of 2. Statistical significance determined by unpaired T-test; (not-significant, ns, $P > 0.05$, *$P<0.05$, ***$P<0.001$, ****$P<0.0001$). Data are presented as $\bar{x} \pm$ SEM. (D-F). Flow plot depicting parasite-specific tetramer$^+$ CD4$^+$ and CD8$^+$ T cells within the brain from isotype and αCD122 treated mice. Quantification of the frequency and total number of parasite-specific CD4$^+$ and CD8$^+$ T cells in the brain as determined by flow cytometry. N=5-10 per group. Pooled data from 2 independent experiments. Statistical significance determined by unpaired T-test; (not-significant, ns, $P > 0.05$, *$P<0.05$). Data are presented as $\bar{x} \pm$ SEM. (G) Quantification of parasite DNA in the brain as determined by RT-qPCR. Statistical significance determined by unpaired T-test; (not-significant, ns, $P > 0.05$). Data are presented as $\bar{x} \pm$ SEM (H) Quantification of the gMFI of CD122 on brain Conventional CD4$^+$ T and CD8$^+$ T as determined by flow cytometry. Statistical significance determined by Two-way Anova and Tukey's multiple correction; (not-significant, ns). Data are presented as $\bar{x} \pm$ SEM. (I) Quantification of the frequency and total number of Foxp3$^+$ Treg cells in the brain from isotype and αCD122 treated mice. N=5-10 per group. 1 representative experiment shown of 2. Statistical significance determined by unpaired T-test; (not-significant, ns, $P > 0.05$). Data are presented as $\bar{x} \pm$ SEM.

Supplemental Figure 3: Efficiency of Foxp3$^{eGFP\ Cre/ERt2+/+}$Ai14$^{Tdtomato+/+}$ mice and whole C3D cleared infected thymus video. (A) Foxp3$^{eGFP\ Cre/ERt2+/+}$Ai14$^{Tdtomato+/+}$ mice were infected with 20 ME49 cysts by I.P.and then treated with a single dose of tamoxifen (8mg), and Treg cell populations were analyzed at day 35 by flow cytometry. (B) Flow plot depicting the frequency of Tdtomato^{+} Foxp3^{+}Treg cells from the thymus, spleen and liver at day 35 after one dose of Tamoxifen. (C). Survival was monitored daily and data presented are from a representative experiment of 2 performed. (D). Whole C3D-cleared thymus from naïve Treg reporter mice was harvested, cleared, and analyzed by immunofluorescence microscopy. 1 representative image depicting the naïve thymus. Box 1 is a representative image of a 40X zoom within the medullary region of the naïve thymus. Single channel images for each representative marker displayed to the right of Box 1 image. (E) Whole C3D-cleared thymus from infected Treg reporter mice was harvested, cleared, and analyzed by immunofluorescence microscopy. Z-stacked images were collected for a depth of ~100um and image sequences across channels as converted into a video file.
